# Enhancing Performance of a Hybrid EEG-fNIRS System Using Channel Selection and Early Temporal Features

**DOI:** 10.3389/fnhum.2017.00462

**Published:** 2017-09-15

**Authors:** Rihui Li, Thomas Potter, Weitian Huang, Yingchun Zhang

**Affiliations:** ^1^Department of Biomedical Engineering, University of Houston Houston, TX, United States; ^2^Guangdong Provincial Work-Injury Rehabilitation Hospital Guangzhou, China

**Keywords:** NIRS, EEG, hybrid BCI, general linear model, principal component analysis

## Abstract

Brain-Computer Interface (BCI) techniques hold a great promise for neuroprosthetic applications. A desirable BCI system should be portable, minimally invasive, and feature high classification accuracy and efficiency. As two commonly used non-invasive brain imaging modalities, Electroencephalography (EEG) and functional near-infrared spectroscopy (fNIRS) BCI system have often been incorporated in the development of hybrid BCI systems, largely due to their complimentary properties. In this study, we aimed to investigate whether the early temporal information extracted from singular EEG and fNIRS channels on each hemisphere can be used to enhance the accuracy and efficiency of a hybrid EEG-fNIRS BCI system. Eleven healthy volunteers were recruited and underwent simultaneous EEG-fNIRS recording during a motor execution task that included left and right hand movements. Singular EEG and fNIRS channels corresponding to the motor cortices of each hemisphere were selected using a general linear model. Early temporal information was extracted from the EEG channel (0–1 s) along with initial hemodynamic dip information from fNIRS (0–2 s) for classification using a support vector machine (SVM). Results demonstrated a lofty classification accuracy using a minimal number of channels and features derived from early temporal information. In conclusion, a hybrid EEG-fNIRS BCI system can achieve higher classification accuracy (91.02 ± 4.08%) and efficiency by integrating their complimentary properties, compared to using EEG (85.64 ± 7.4%) or fNIRS alone (85.55 ± 10.72%). Such a hybrid system can also achieve minimal response lag in application by focusing on rapidly-evolving brain dynamics.

## Introduction

Brain-Computer Interface (BCI) systems, which use cortical activity to control external devices, have shown promising potential for multiple applications (Wolpaw et al., 2002). One of the main focuses of current BCI-related research is increasing the efficiency of real-time reactions while using a convenient setup that minimizes the burden on the user. Considering factors like setup cost and time resolution is therefore essential when choosing measurement modalities for a BCI study. BCI systems can be either invasive or non-invasive (Blankertz et al., 2007; Miller et al., 2010; Brunner et al., 2011), though non-invasive BCIs are usually preferable since they incur neither the expenses nor safety risks of electrode implantation.

Over the past few decades, different non-invasive methods, including Electroencephalography (EEG) (Salvaris and Sepulveda, 2010; Choi, 2013; Rejer, 2015), functional Near-Infrared Spectroscopy (fNIRS) (Coyle et al., 2004, 2007; Khan et al., 2014; Naseer et al., 2016), functional Magnetic Resonance Imaging (fMRI) (Lee et al., 2009; Sorger et al., 2009), and Magnetoencephalography (MEG) (Waldert et al., 2008), have been extensively explored. Each modality has its own strengths and limitations, so it falls to the experimenter to select an appropriate method with high efficiency and low cost. Current practice then shows that EEG and fNIRS are considered the leading non-invasive BCI modalities due to their modest costs and practicality (von Lühmann et al., 2015; Lin and Hsieh, 2016; Khan and Hong, 2017).

Electroencephalography (EEG) is a non-invasive brain imaging technique that uses scalp electrodes to measure the voltage fluctuations induced by the mass electrical activity of neurons. While this technique provides a direct measurement of brain activity, EEG systems can be sensitive to noise. In particular, EEG is highly vulnerable to motion artifacts, which would inhibit BCI accuracy in a practical setting (Yuan et al., 2008; He et al., 2011).

Functional near-infrared spectroscopy (fNIRS) is a non-invasive optical imaging technique that usually utilizes two distinct wavelengths (between 600 and 1000 nm) to measure the concentration changes of oxygenated hemoglobin (HbO) and deoxygenated hemoglobin (HbR) that are coupled with the metabolic activity of neurons in the outer layers of the cortex. These measures have proven to be similar to the blood oxygen level dependent (BOLD) response obtained by fMRI (Ferrari and Quaresima, 2012; Boas et al., 2014), though the fNIRS system is portable and features a much higher sampling rate. The main limitations of fNIRS-based BCI lie in the long delay of the hemodynamic response, which takes 4–6 s to reach its maximum amplitude, and the limited penetration depth of infrared light, which limits detection to outer cortical regions. These result in a poor temporal efficiency which is considered to be a major obstacle for a real-time fNIRS-based BCI application (Naseer and Hong, 2015). In light of these weaknesses, fNIRS does feature enhanced spatial accuracy—hemodynamic signals do not spread between channels like electrical signals—and a remarkable resilience to motion artifacts, making the technique useful for mobile and prosthetic applications.

The complimentary individual properties of EEG and fNIRS have led to active investigations of the benefits of integrated EEG and fNIRS in a number of BCI studies (Fazli et al., 2012; Putze et al., 2014; Buccino et al., 2016). In general, integrated EEG-fNIRS approaches offer various benefits over single-modality methods by capitalizing on their individual strengths; EEG provides favorable temporal resolution (about 0.05 s), while fNIRS offers better spatial resolution (about 5 mm) and is robust to noise (Nicolas-Alonso and Gomez-Gil, 2012; Waldert et al., 2012). Secondarily, EEG and fNIRS signals are associated with different aspects of cortical activity, providing a built-in validation for identified activity. Measurements obtained from each of these two modalities thereby provide complementary information and can be used to enhance the performance of BCIs.

In hybrid EEG-fNIRS BCI applications, the main challenge is how to improve the classification accuracy while reducing the complexity of system and improving response time (Naseer and Hong, 2015; Shin et al., 2017; Zafar and Hong, 2017). Since Fazli et al. (2012) showed that BCI performance in a binary motor task can be enhanced by incorporating EEG features with those derived from the fNIRS signals, hybrid EEG-fNIRS BCIs have become a major research focus. These multimodal BCIs have shown enhanced classification accuracy in a variety of tasks, including mental arithmetic (MA), hand rotations, and movements (Naito et al., 2007; Abibullaev and An, 2012; Yin et al., 2015). However, some methodological limitations remain unsolved. For example, most hybrid EEG-fNIRS systems have relied on principle component analysis (PCA) or common spatial pattern (CSP) methods to transform the original data and select the components with largest discriminability between the two target classes (Blankertz et al., 2008; Li et al., 2015). As a result, multiple channels—usually all available channels from both hemispheres—are required to perform feature extraction, classifier training, and classifier testing. This dramatically increases both computational and systemic costs and reduces the stability of the system setup. Furthermore, the purpose of integrating EEG and fNIRS in a BCI study should be to achieve a true multimodal integration that accentuates the favorable properties of each individual approach (Al-Shargie et al., 2016). In particular, the spatial information of fNIRS could be further exploited to enhance hybrid EEG-fNIRS studies. Unfortunately, most hybrid BCIs simply process the signals separately and combine two groups of features for classification. Finally, although high classification accuracy has been achieved (Mihara and Miyai, 2016), the temporally slow hemodynamic response and wide time window used for feature extraction remain major issues associated with the use of fNIRS for BCI applications (Naseer and Hong, 2015).

In this study, we aimed to perform a binary classification of left and right hand movements in a hybrid EEG-fNIRS BCI system using signals obtained from the motor cortex. A channel selection criterion based on the general linear model (GLM) was proposed. The early information from the selected EEG channels was extracted using a short time window (0–1 s) while the initial dip (0–2 s) of the hemodynamic response was captured from the selected fNIRS channels. To our knowledge, this is the first hybrid EEG-fNIRS-based BCI study to take advantage of the spatial information of fNIRS for channel selection and apply the early temporal information of both modalities to enhance the transfer rate of the system while maintaining a decent performance.

## Materials and methods

### Participants

Eleven healthy, right-handed subjects (*n* = 11, male, 25.5 ± 3.2 years) participated in this experiment. The experiment was approved by the local ethics committee (Guangdong Provincial Work Injury Rehabilitation Center, China), and performed in accordance with the Declaration of Helsinki. Each subject was fully informed about the purpose of the research and provided written, informed consent prior to the start of the experiment. No participants had any history of neurological or psychiatric disorders or disease. No participants had any previous experience with the experimental task and all were naive to the BCI.

### Experiment paradigm

**Figure 1:**
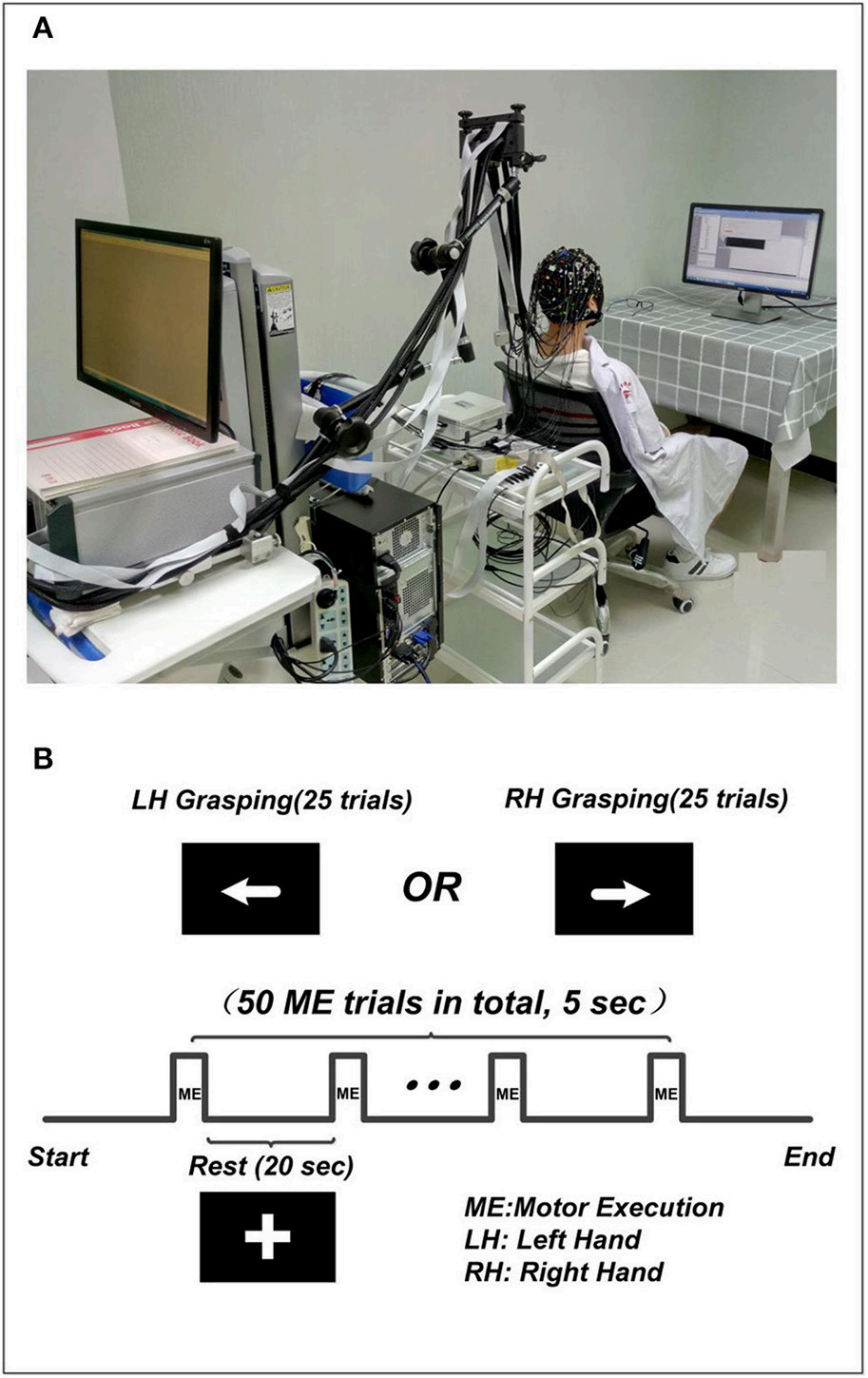
The experiment setup. **(A)** The environment of concurrent EEG-fNIRS measurement. The subject included in the figure was provided written, informed consent for the publication of this figure. **(B)** The paradigm used in the experiment. The “+” indicates the rest condition, the left arrow indicates left hand grasping task, and the right arrow indicates the right hand grasping task.

The experiment was performed in a confined room to reduce any environmental disturbances. During the experiment, subjects were seated in a comfortable chair and asked to remain still and relaxed. Subjects received visual instruction through a screen placed 1 m in front of their eyes (Figure 1A). The motor execution paradigm used in the experiment consisted of 50 randomized trials of left and right hand grasping tasks (25 trials for each hand movement). Each trial started with 20 s of rest, indicated by a “+” symbol, followed by 5 s of motor execution, in which an arrow was shown pointing either left or right, as shown in Figure 1B. Subjects were asked to squeeze a rubber ball with the corresponding hand for the entire duration that the arrow stimulus was shown.

### System setup

**Figure 2:**
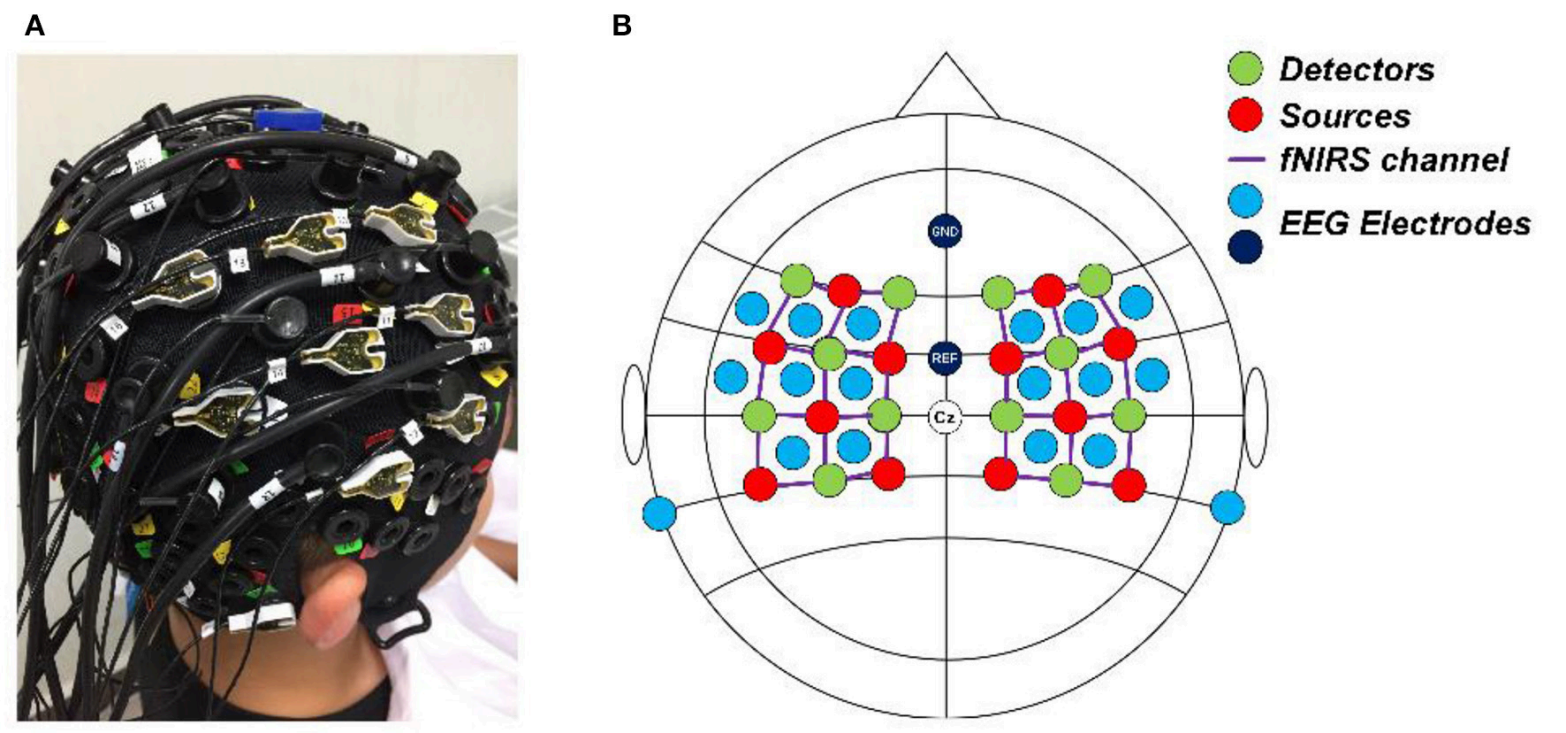
**(A)** Real photo of a subject wearing the cap completely mounted with EEG electrodes, fNIRS sources and detectors. **(B)** The configuration of the EEG electrodes and fNIRS optodes on the cap. Red circles denote the sources of fNIRS, green circles denote the detectors of fNIRS, the purple lines denote the fNIRS channels, and light blue and dark blue circles denote the EEG electrodes.

A concurrent EEG and fNIRS measurement setup was employed in this study. EEG signals were recorded at 500 Hz using a BrainAmp DC EEG recording system (Brain Products GmbH, Germany). Sixteen EEG electrodes were placed on the scalp over the left and right motor cortices (FFT7h, FFC5h, FFC3h, FFT8h, FFC6h, FFC4h, FTT7h, FCC5h, FCC3h, FTT8h, FCC6h, FCC4h, CCP5h, CCP3h, CCP4h, and CCP6h). Two EEG electrodes were attached on both mastoids, the average of their signals was used as re-reference signal in preprocessing raw EEG data. FNIRS signals were recorded simultaneously using a NIRScout system (NIRx Medizintechnik GmbH, Germany) with 12 sources and 12 detectors. The inter-optode distance was 3 cm and a total of 34 fNIRS channels were equidistantly distributed throughout the motor cortex areas. The wavelengths used for oxy- and deoxy- hemoglobin detection were 760 and 850 nm, respectively. The fNIRS signals were acquired at a sampling rate of 7.81 Hz. A schematic illustration of the location of EEG electrodes and fNIRS channels is shown in Figure 2.

### Data preprocessing

Raw EEG signals of all channels were first re-referenced by subtracting the average of two EEG channels on both mastoids. Since the valuable EEG information related to motor function is usually related to frequencies below 40 Hz (Pfurtscheller and Neuper, 2001), raw EEG signals were first down-sampled to 250 Hz and filtered from 1 to 45 Hz using a 3rd order Butterworth band-pass filter. Single-trial EEG data was segmented from 2 s prior to the onset of movement instruction (baseline: −2–0 s) to 5 s after the onset (execution: 0–5 s), resulting in 25 segmented trials for each hand movement. Baseline correction was performed by subtracting the mean value of individual baseline interval from its corresponding segmented trial.

To process the fNIRS signal, the concentration changes of hemoglobin (HbO and HbR) were computed using the Modified Beer-Lambert Law (the differential path length factors for the higher (850 nm) and lower (760 nm) wavelengths were 6.38 and 7.15, respectively) (Scholkmann et al., 2014). A 4th order Butterworth band-pass filter was applied from 0.01 to 0.2 Hz to remove artifacts, including cardiac interference (0.8 Hz) and respiration (0.2–0.3 Hz) (Zhang et al., 2005). In addition, spline interpolation was performed to remove any motion artifact contamination from the fNIRS signal (Scholkmann et al., 2010). Single trial fNIRS data was segmented from 5 s prior to the onset of movement instruction (baseline: −5–0 s) to 20 s after the onset (execution: 0–20 s), creating fNIRS trials that directly correspond to those obtained through EEG segmentation. The mean value of each baseline signal was subtracted from associated execution task.

### Channel selection

Before features can be extracted, it is essential that appropriate channels are selected if a BCI system is to achieve favorable accuracy with minimal complexity. Previous work has suggested different approaches for selecting the most representative channels or signal components for classification, including common spatial patterns (Blankertz et al., 2008), bundled-optode-based approaches (Nguyen and Hon, 2016), and channel-averaging approaches (Khan and Hong, 2015). A main goal of this paper is to use the spatial information from fNIRS to identify the single fNIRS channel and EEG channel on each hemisphere that yields the most significant differences between the binary motor tasks, which will enable increased classification accuracy with as few channels as possible. Here, the general linear model (GLM), a well-known and widely used method that fits the expected hemodynamic response to the measured fNIRS signal, was applied to show the channels that yield the largest contrast between the two classes (Penny et al., 2011).

Both HbO and HbR concentration changes reflect changes in the hemodynamic response, though it has been suggested that HbO is a more sensitive indicator in fNIRS studies (Holper et al., 2009). Therefore, HbO was adopted in the GLM analysis of the present study.

The GLM model is given by:

$$\begin{array}{l} Y\;=X\beta +\varepsilon \end{array}$$

where *Y* is an N × M matrix of measured data (where N denotes the number of data points and M denotes the number of fNIRS channels), *X* is an N × L design matrix (where L denotes the number of the conditions, including the tasks and any term that is considered as a source related to the variance of the data). β is a L × M matrix of regression coefficients to be estimated where L is associated with the number of the conditions and the value of β reflects the magnitude of the condition-evoked brain response. Finally, ε is an N × M matrix of residual error. In this present study, β is a 3 × M matrix assigned with three conditions, where the first row indicates the left hand movement, the second row indicates the right hand movement, and the third row is a constant term on all channels.

The regression coefficient β and the residual error ε can be tested through a one-sample *t*-test to identify the channels with *t*-values that represent a significant contrast between the two motor execution tasks. This *t*-value is calculated by:

$$\begin{array}{l} t=\frac{c^{T}*\beta }{\sqrt{\varepsilon^{2}c^{T}{\left(X^{T}X\right)}^{-1}c}\;} \end{array}$$

where *c* is the contrast vector, which determines the contrast between specific conditions.

**Figure 3:**
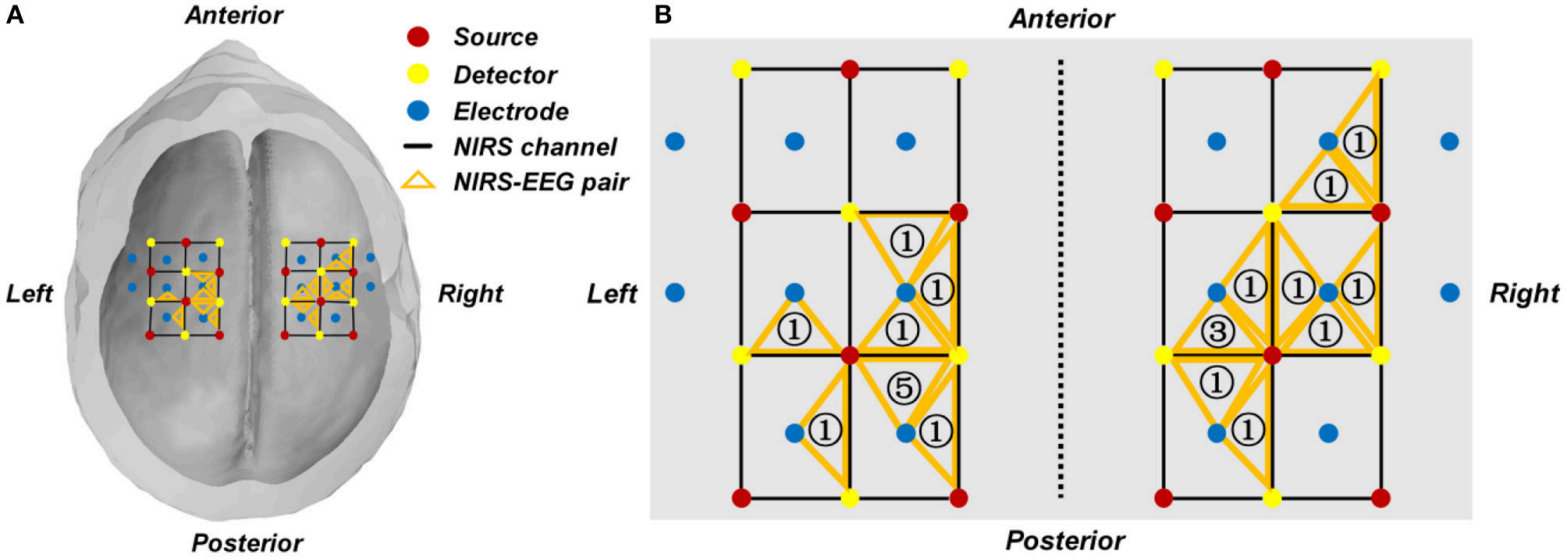
**(A)** Group-wise location summary of the selected EEG and fNIRS channels for all subjects. **(B)** Zoom-in view of the group-wise summarized location. An orange triangle represents a pair of selected fNIRS channel and their corresponding EEG channel. The number in the triangle represents the number of subjects whose selected channel is located at the given area.

In our study, the following criterion was used to select the EEG channel and fNIRS channel of interest. First, the regression coefficient β of each individual fNIRS channel was estimated through the GLM, from which a group of channels with *t*-values that represent a significant contrast between the two motor execution tasks were selected as candidate channels. For each hemisphere, an fNIRS channel that yielded the highest *t*-value among those candidate channels was selected. One EEG channel, which was adjacent to the chosen fNIRS channel, was selected for classification. Therefore, the two EEG channels were selected according to the two fNIRS channels with the greatest discriminatory potential. The subject-specific locations of the selected channels are summarized and shown in Figure 3 and Table 1.

**Table 1:** The overall selected fNIRS channels and EEG electrodes of all subjects.

**Subject no**.	**Left hemisphere**		**Right hemisphere**	
	**NIRS**	**EEG**	**NIRS**	**EEG**
1	7	FCC3h	31	CCP4h
2	12	CCP3h	28	CCP4h
3	12	CCP3h	24	FFC6h
4	15	CCP3h	26	FCC6h
5	12	FCC3h	28	FCC4h
6	11	FCC5h	28	FCC4h
7	12	CCP3h	22	FFC6h
8	12	CCP3h	26	FCC4h
9	12	CCP3h	28	FCC4h
10	14	CCP5h	29	FCC6h
11	10	FCC3h	27	FCC6h

### EEG feature extraction

In order to extract the features associated with early temporal information, EEG data from 0 to 1 s (0 s denoting the onset of the stimuli) was segmented out from the selected channels, resulting in a 1 s-long time window of EEG data with 250 points for each trial. The discrete wavelet transform (DWT) was then employed to decompose the segmented single trial EEG data (Samar et al., 1999), DWT is a technique that decomposes time series data of each selected EEG channel into a number of layers. In each layer the signal is filtered with a quadrature mirror filter (a low-pass filter and a high-pass filter). The output of each layer is a series of detail coefficients (from the high-pass filter) and approximation coefficients (from the low-pass filter). In this study we assumed that the wavelet approximation coefficients from the output of the last DWT layer contained the main power of the event-related oscillation in brain activity (Subasi, 2007), which can be used for the discrimination of left and right hand movements. Here, the segmented signals of the selected EEG channels were decomposed with a 4-layer “Symlet” wavelet, resulting in 22 approximation coefficients for each trial. Then all approximation coefficients of the selected EEG channels were combined into a 44-dimensional EEG feature set (22-dimensional × 2 channels) for the single trial classification of the left and right hand movements.

### fNIRS feature extraction

The peak information from the HbO and HbR signals has been widely used in many fNIRS-based BCI studies (Naseer and Hong, 2015). However, the inherent delay of the hemodynamic response impedes the efficiency of a real-time fNIRS-based BCI application.

The hemodynamic feature of interest in the current study is known as the initial dip—a metabolically-linked phenomenon wherein HbO concentration decreases slightly or HbR concentration increases slightly 0–2 s after the presentation of stimuli (Frostig et al., 1990). This fluctuation is considered to be the early and rapid metabolism of blood-borne oxygen by the responding population of neurons, occurring before the main activity-coupled vascular response. Though the initial dip has a relatively low amplitude, Zafar et al. have shown that detecting and classifying the initial dips is feasible with fNIRS (Zafar et al., 2016). As a result of their rapid evolution in the face of stimuli the initial dip information was extracted for classification in this study.

Prior to the extraction of initial dip information, principal component analysis (PCA) was performed to further remove any artifacts remaining in the preprocessed fNIRS signal. In this manner, the N-trial fNIRS data set from the selected channel was transformed into N linearly uncorrelated components known as principal components, ordered by the amount of variance of the original data that each component accounts for. The application of PCA to filter the multi-trial fNIRS data within a channel assumes that the event-evoked hemodynamic response is the main component across all trials. This means that the hemodynamic response provides the dominant contribution to the variance of the fNIRS data and implies that the first several principal components will be similarly linked to the expected event-evoked hemodynamic response.

The PCA filtration is given by:

$$\begin{array}{l} Y=E*X \end{array}$$

where *X* is the N × M data matrix (in which N denotes the data points of each trial and M denotes the number of trials), *E* is the eigenvector matrix with the dimensions N × N, and *Y* is the N × M matrix consisting of the N uncorrelated principal components. By keeping the first R components with the largest variances and removing the remaining components, the original data X can be reconstructed by:

$$\begin{array}{l} X_{recon}=Y_{new}*E_{new}^{T} \end{array}$$

where *X*_*recon*_ is the N × M filtered data, *E*_*new*_ is the new eigenvector matrix with dimension N × R, and *Y*_*new*_ is the N × R matrix consisting of the R uncorrelated principal components.

In our study, all trials of each hand movement were filtered by PCA with the first component accounting for approximately 70% of the variance of the data set. Then the mean values of the HbO and HbR fluctuations within the 0–2 s interval were computed for each trial, resulting in a 4-dimensional fNIRS feature set (2 mean values (HbO + HbR) × 2 channels) for the single trial classification of the left and right hand movements.

### Classification

Prior to the classification, we constructed three different feature sets: EEG-only feature set, fNIRS-only feature set, and a hybrid feature set (EEG + fNIRS). The EEG-only feature set contained 44 approximation coefficients obtained from the selected EEG channels for each trial, while the fNIRS-only feature set contained 4 hemodynamic features (mean values of HbO and HbR of the two selected fNIRS channels) for each trial. Then all single trial features in both modalities were respectively normalized and rescaled between 0 and 1. The hybrid feature set was formed as a 48- dimensional feature vector for each trial, which contained the normalized EEG features (44 dimensional) and fNIRS features (4 dimensional). In summary, the dimensions of hybrid feature vectors were 48 features × 25 trials for either a left or right hand movements.

**Figure 4:**
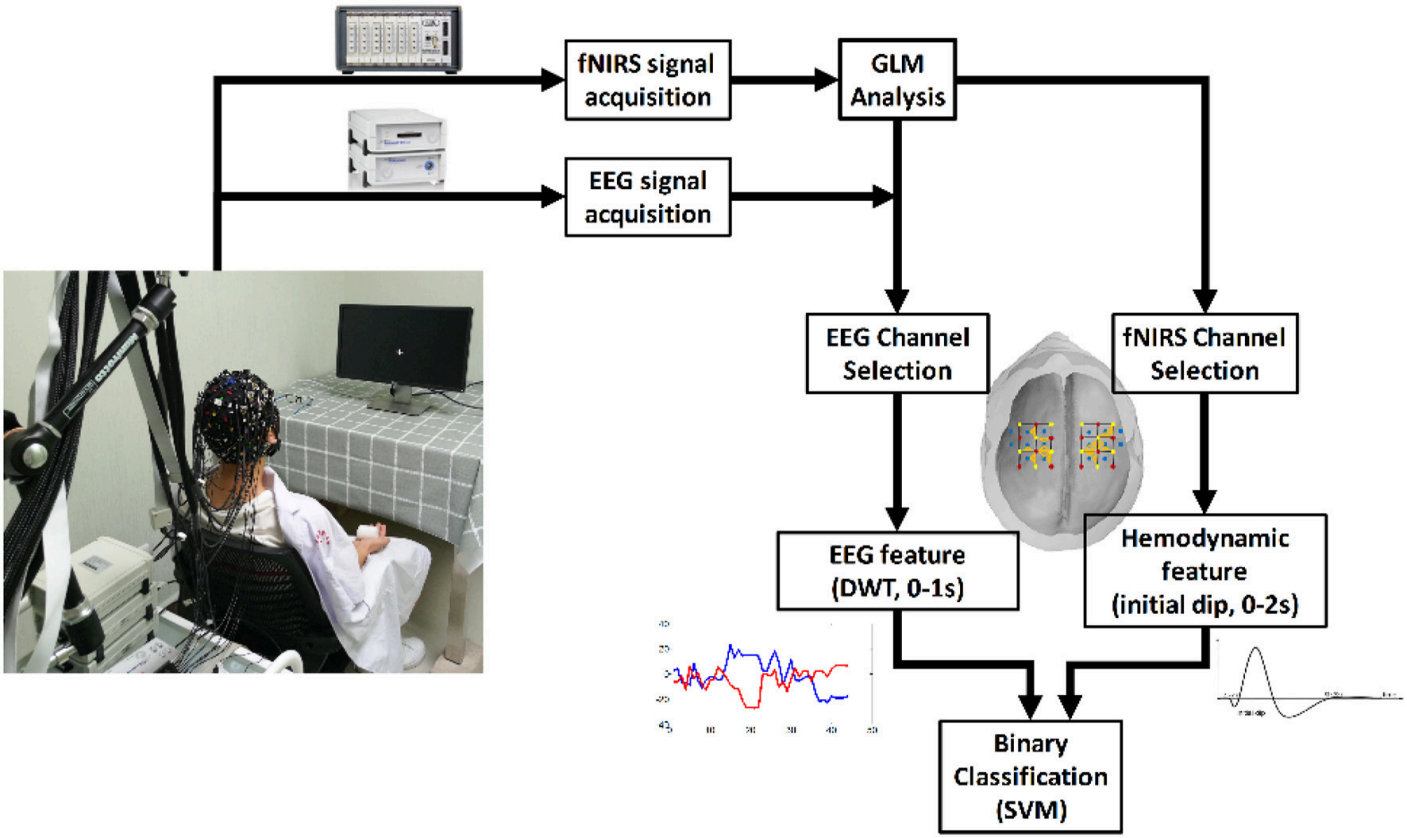
The flow chart of the study.

A support vector machine (SVM) was applied to perform the classification of the two-hand motor execution for each individual subject. The goal of SVM is to construct a hyper-plane that maximize the margins between the classes by minimizing the cost function (Drucker et al., 1997). In this study a SVM toolbox named “LIBSVM” was employed to train the SVM classifier and perform the prediction (Chang and Lin, 2011). In particular, a Radial Basis Function (RBF) kernel which works under both linear and nonlinear situations was applied with default parameters (penalty parameter *C* = 1, γ = 1/number of features). As the obtained feature set was small (25 trials in total for each motor task), the Leave-One-Out cross-validation (LOOCV) method was utilized by randomly selecting one trial as a testing set and using the remaining 24 trials as the training set to train a classifier for prediction until all trials were tested. The classification accuracy for each subject was calculated as the ratio between the number of correct predictions and the total number of predictions. Classification was performed separately using three kinds of feature sets for comparison; an EEG-only feature set, an fNIRS-only feature set, and a hybrid feature set (EEG + fNIRS). A flowchart is presented in Figure 4 to describe the study design.

## Results

Figure 3A shows a summarized mapping of the EEG and fNIRS channels selected from each subject for classification based on the GLM results. Each triangle indicates an EEG-fNIRS pair of selected channels. The number in the orange triangle represents the number of subjects whose selected channel is located at the given area, as shown in Figure 3B. The selected fNIRS channels and EEG channels of each individual subject are shown in Table 1.

**Figure 5:**
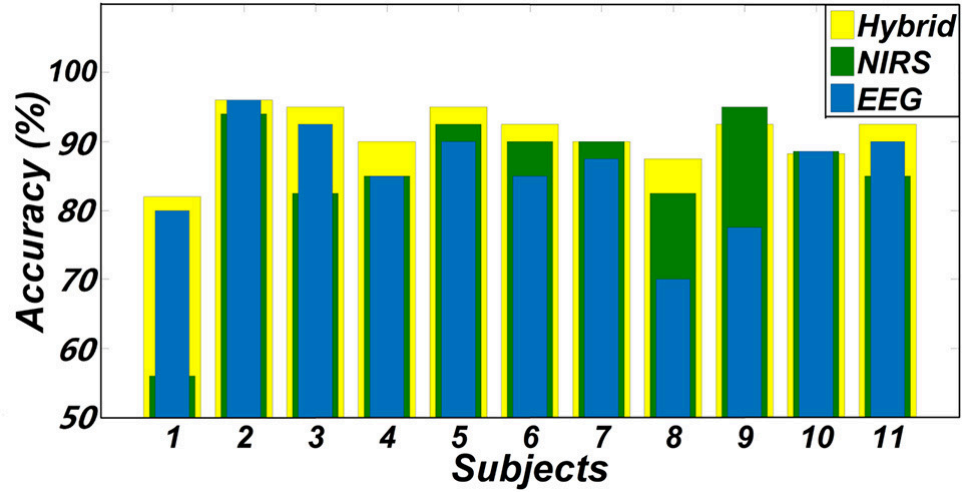
Classification accuracies of two hand movements obtained from three feature sets (EEG + fNIRS, EEG-only, and fNIRS-only).

**Figure 6:**
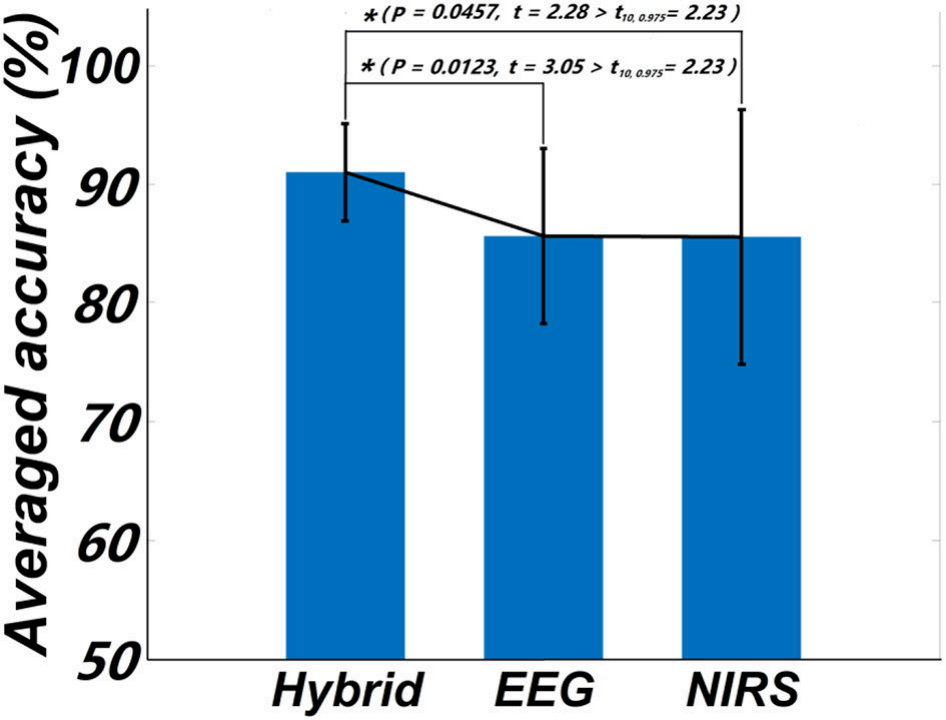
Statistical plot of the classification accuracies obtained from the three feature sets, respectively. The asterisk “^*^” indicates a significant difference (*p* < 0.5, t_10, 0.975_ denotes the critical value with 10 degree of freedom and significant level = 0.05).

One goal of our study was to comparatively evaluate the classification reliability of the features extracted from EEG, fNIRS, and EEG + fNIRS based on the results of the GLM. To do this, we performed a single-trial classification of the left vs. right motor execution task. Classification accuracies obtained from each subject by the three different feature sets can be seen in Table 2. A classification accuracy of 100% would indicate that the two motor tasks are perfectly separable, while a classification accuracy of 50% would represent the poor performance of a random classifier in the context of the binary classification task. Figure 5 shows the histogram plot of all classification results, with the overall classification accuracies of all three feature sets exceeding 85%. Specifically, the average accuracy of the EEG-only feature set (85.64 ± 7.4%) slightly outperformed the fNIRS-only feature set (85.55 ± 10.72%). The best performance, however, was achieved from the hybrid EEG-fNIRS feature set (91.02 ± 4.08%), providing an improvement in the classification accuracy and minimizing the standard deviation. To examine how significantly the hybrid feature set outperformed the single modality, paired *t*-test was applied to test the classification results obtained by the three different feature sets. Prior to the paired *t*-test, the W/S test was firstly performed to test the normality of the obtained classification accuracies, which is the prerequisite of paired *t*-test analysis (Kanji, 1993). The result revealed that all the accuracies were normally distributed at a significance level of 0.05 [q_EEG_ = 3.5124, q_fNIRS_ = 3.6365, q_Hybrid_ = 3.4275, q_critical_ = (2.74 3.80)]. The statistical results of paired *t*-test are shown in Figure 6. It can be observed that the classification performance based on the hybrid feature set was significantly improved over classification based on EEG-only features (*P* = 0.0123) and classification based on fNIRS-only features (*p* = 0.0457) as well.

**Table 2:** Summary of SVM classification accuracies for feature sets of NIRS-only (HbO + HbR), EEG-only and hybrid (EEG + fNIRS).

**Subject no**.	**Accuracy (%)**		
	**EEG**	**fNIRS**	**EEG + fNIRS**
1	80.0	56.0	82.0
2	96.0	94.0	96.0
3	92.5	82.5	95.0
4	85.0	85.0	90.0
5	90.0	92.5	95.0
6	85.0	90.0	92.5
7	87.5	90.0	90.0
8	70.0	82.5	87.5
9	77.5	95.0	92.5
10	88.6	88.6	88.2
11	90.0	85.0	92.5
Mean (%)	85.64	85.55	91.02
Std. (%)	7.40	10.72	4.08

## Discussion

Multi-modal imaging has been reported to improve classification accuracy over unimodal methods (Fazli et al., 2012). In this paper, we attempted to achieve the highly accurate and computationally efficient classification of a binary motor execution task using a hybrid BCI design. This was performed through the selection of singular hemispheric EEG and fNIRS channels and the application of rapidly-evolving temporal features from both modalities. The results indicated that the multi-modal fNIRS-EEG approach significantly improved the performance over that of unimodal alone, yielding an average accuracy of 91.02 ± 4.08% and proving the suitability of the hybrid approach for binary motor execution tasks.

Channel selection plays a crucial role in the design and application of a BCI system, especially with respect to the number and the location of the selected channels. For the classification of motor execution tasks, it is quite common to utilize multiple channels from the C3 and C4 areas (Fazli et al., 2012; Buccino et al., 2016). These methods, however, might not be able to minimize the variation from subject to subject, as identical channels may align with different brain regions. Although recent studies have investigated the efficiency of different channel selection criteria (Blankertz et al., 2008; Khan and Hong, 2015; Nguyen and Hon, 2016), few efforts have been made to optimize the number and location of these channels. A previous fNIRS study proposed a selection criterion based on high *t*-value channels from the auditory cortex during the classification of four sound categories (Hong and Santosa, 2016). This method, however, still relied on multiple channels with no noticeable improvement in performance. In this study, we only made use of single EEG and fNIRS channels from each hemisphere with the highest *t*-value based on the GLM results for classification. Here we attempted to capitalize on the spatial information from fNIRS, a valuable advantage of fNIRS technology, to ensure that the most effective channels were chosen for feature extraction and classification. As such we might be able to reduce the complexity of a BCI system and minimize the burden on the user. Table 3 summarized the results of recent EEG-fNIRS-based BCI studies using different numbers of channels and different lengths of time windows in motor execution or imagery tasks. It is noticeable that although very few channels were selected in our study, the average accuracy among all subjects tended to be slightly lower but still comparable with previous studies reported in Table 3. Results demonstrated that it is feasible to take advantage of the high spatial resolution offered by fNIRS to select channels for classification, to therefore reduce the channel number and the complexity of the BCI system while maintaining desirable performance.

**Table 3:** Summary of recent EEG-fNIRS-based BCI studies using a motor execution or imagery task.

**Reference**	**Task**	**Channel no**.	**Accuracy**
Fazli et al., 2012	Hand griping	24 fNIRS channels	93.2%
	(Motor execution)	37 EEG electrodes	
Khan et al., 2014	Finger taping	12 fNIRS channels	94.7%
	(Motor execution)	8 EEG electrodes	(Motion vs. Rest)
Koo et al., 2015	Hand grasping	8 fNIRS channels	88%
	(Motor imagery)	6 EEG electrodes	
Yin et al., 2015	Hand clenching	24 fNIRS channels	89%
	(Motor imagery)	21 EEG electrodes	
Buccino et al., 2016	Arm raising and hand griping	34 fNIRS channels	72.2%
	(Motor execution)	21 EEG electrodes	(Right vs. Left)

In addition to the classification performance, the proposed channel selection criterion applied the spatial information from fNIRS to the selection of both the EEG and fNIRS channels, establishing a connection between the two modalities rather than performing the separated channel selection performed in previous studies (Fazli et al., 2012; Buccino et al., 2016; Khan and Hong, 2017). The reliability of this proposed method was validated by the favorable classification performance shown in Figure 5, where all of the average classification accuracies using the three different feature sets exceeded 85%. It is noteworthy that, while all selected channels were located within the motor cortex, the exact channel of interest varied by subject (Figure 3). This shows that the proposed channel selection method was able to identify appropriate, subject-specific channels according to the GLM results, minimizing any error from potential variation in channel positions. The mapping results therefore emphasize the importance of selecting customized channels from each individual subject instead of simply choosing motor-related channels—like C3 or C4—for motor task classification. One limitation of this criterion is that multiple channels are needed to cover the targeted area and obtain the GLM results during the training session. However, even if the selection of appropriate channels (EEG and fNIRS) requires extra channels during the training session, selection would enable a simplified practical BCI system that can be adjusted and tailored to fit each individual user.

One concern of the GLM-based channel selection is the reliability of applying this approach. The performance of the GLM may be subject to artifacts that contaminate the raw data, such as motion artifacts, low frequency trends, and serial correlations (Hu et al., 2010). It is therefore necessary to ensure that data is appropriately processed and that these artifacts are effectively removed before to applying the GLM. In the present study, to ensure that reliable GLM results could be obtained, spline interpolation and bandpass filtration were applied to remove motion artifacts and low frequency trends. As our study solely relies on a single channel chosen on each hemisphere, channels contaminated by other artifacts that could not be removed through preprocessing methods would possibly decrease the performance of classification. This may have been the case for subject 1, who consistently showed lower classification accuracies, regardless of the feature set (Figure 5, Table 2). More effective preprocessing algorithms can be furthered explored in our future work to improve the stability of the GLM and alleviate this issue. It should be noted that, while unimodal classification may have been poor in this case, the hybrid combination revealed the potential to stabilize the classification performance with a higher mean accuracy and smaller standard deviation (Figure 6, Table 2). Apparently, the inclusion of the different information measured by EEG and fNIRS is beneficial to the robustness of the BCI. Another concern of the GLM-based channel selection method is whether the proposed method is superior to the conventional approaches, which was not done in this very initial study. Since few studies considered to apply the GLM-based method in a hybrid EEG-fNIRS-based BCI study, a comprehensive comparison between the proposed method and the conventional approaches, including different features, channels of different numbers and time windows of different lengths, would be actively investigated in our future work.

**Figure 7:**
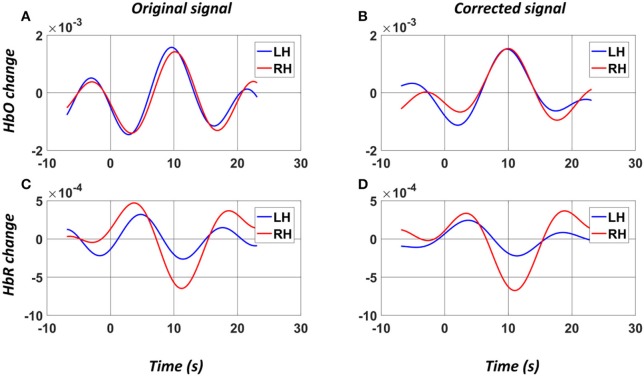
Example (Subject 2) of the average HbO and HbR signals of a selected channel on left hemisphere before **(A,C)** and after **(B,D)** the PCA denoising. The “0” denotes the onset of the stimuli. LH: Left Hand (blue); RH: Right Hand (red).

EEG-based BCIs have been reported to yield superior temporal results in real-time BCI applications (Nicolas-Alonso and Gomez-Gil, 2012). Recently, fNIRS-based BCIs have also been developed that show favorable classification rates by using different combinations of features and various classifiers (Naseer and Hong, 2015). These fNIRS-based BCIs, however, are not yet viable as an alternative to EEG-based BCIs; the most reliable feature of fNIRS is the HbO peak information, which shows a long delay in the response to stimuli (Naseer and Hong, 2015). In this study, we aimed to enhance the response efficiency of a hybrid system while maintaining favorable accuracy. This was performed by focusing on the initial dip of the hemodynamic response, which has been proven to be a potential feature for fNIRS-based BCI application (Zafar and Hong, 2017). Generally it is difficult to detect the initial dip due to its short duration and high sensitivity to low frequency artifact (e.g., Mayer wave). In order to obtain a clean initial dip in single-trial fNIRS signal, a PCA-based algorithm was employed to extract the main component, which was considered the true hemodynamic response associated with the motor execution task. In the present study, we selected the first principal component, which accounted for over 70% of the total variance of the original signal. Figure 7 shows the original averaged HbO and HbR signals as well as the PCA-corrected HbO and HbR signals in a selected channel on left hemisphere of a subject. It could be observed that while the original HbO signals induced by two hand movements were quite similar, the PCA-corrected signals clearly showed a difference in the initial dips of the HbO signals, which can be extracted for the binary class classification. And the results showed that this was sufficient to achieve a high classification accuracy. In particular, the lofty classification accuracies obtained by the fNIRS-only classifier (85.55 ± 10.72%) as well as from hybrid classifier (91.02 ± 4.08%) demonstrated the effectiveness of the initial dip in discriminating the binary motor tasks. By applying a 0–2 s time window to the fNIRS signal, it was observed that the addition of fNIRS features significantly enhanced the performance of the EEG-based BCI without significantly increasing the time delay, demonstrating the advantage of a hybrid EEG-fNIRS system and showing that early temporal features can be used to create a faster and more stable BCI system, which overcomes the problem in Fazli's study (Fazli et al., 2012).

A secondary limitation of our study lies in the configuration of the EEG electrodes and fNIRS optodes, where the EEG electrodes were surrounded by the fNIRS channels, as shown in Figure 2. Although we chose EEG electrodes that were close to the selected fNIRS channels, placing the EEG electrodes on the surface pathways of the fNIRS channels may optimize the channel configuration and enhance the physiological consistency between the EEG and fNIRS channels. This problem may be addressed by using a customized cap in the future.

## Conclusion

In this study, a hybrid EEG-fNIRS configuration for binary motor task classification was proposed. Singular EEG and fNIRS channels were selected from the motor cortex of each hemisphere based on the general linear model. Early temporal information from the EEG and fNIRS signals were extracted for classification using a SVM. The high accuracy and efficiency of classification results are encouraging and suggest the integration strategy developed in this study as a promising approach to develop a high-performance BCI system.

## Author contributions

RL conceived this study and contributed to experimental design, data analysis and paper writing. TP and WH contributed to subject recruitment, result interpretation and paper revision. YZ contributed to study design, data analysis, result interpretation and paper writing.

### Conflict of interest statement

The authors declare that the research was conducted in the absence of any commercial or financial relationships that could be construed as a potential conflict of interest.
